# Radiostereometric analysis of the initial stability of internally fixed femoral neck fractures under differential loading

**DOI:** 10.1002/jor.24150

**Published:** 2018-10-25

**Authors:** Sami Finnilä, Niko Moritz, Niko Strandberg, Jessica J. Alm, Hannu T. Aro

**Affiliations:** ^1^ Orthopedic Research Unit, Department of Orthopedic Surgery and Traumatology Turku University Hospital and University of Turku Turku Finland

**Keywords:** radiostereometric analysis, femoral neck fracture, internal fixation, fracture healing

## Abstract

We examined the feasibility of radiostereometric analysis (RSA) in the assessment of the initial stability of internally fixed femoral neck fractures. The study included 16 patients (mean age 73 years). During surgery, multiple RSA‐beads were inserted on both sides of the fracture. Radiographs for RSA were taken in the supine position within the first 3 days and 6, 12, 24, and 52 weeks after surgery. To detect any inducible motion at the fracture‐site, radiographs for RSA were taken with the patient resting or applying a load through the fracture. Fracture loading was achieved by the patient pressing the ipsilateral foot as much as tolerated on a force plate while providing a counterforce through both hands. Micromotion exceeding the precision values of RSA (≥0.3 mm for the translation vector and/or ≥1.2 degrees for the rotation vector) was considered significant. Permanent three‐dimensional fracture‐site displacement was also recorded. Voluntary loading induced fracture‐site micromotion, which exhibited a dichotomous distribution. In patients with uncomplicated fracture union, inducible micromotion was detectable only at baseline—if at all. Conversely, fractures that developed a nonunion were characterized by the continuation of inducible micromotion beyond baseline. Permanent fracture‐site displacement was, on average, nearly an order of magnitude greater than the inducible micromotion. Fracture unions were characterized by the cessation of permanent fracture‐site displacement by 12 weeks. Nonunions presented as outliers in permanent fracture‐site displacement. Large‐scale studies are warranted to evaluate whether the detection of inducible micromotion beyond baseline could serve as an indicator of insufficient fixation stability. © 2018 The Authors. *Journal of Orthopaedic Research*® Published by Wiley Periodicals, Inc. on behalf of the Orthopaedic Research Society.

Fractures of the femoral neck are associated with a substantial risk of incomplete recovery^1^, ^2^ and a high cost to society.^3^ The proper selection of treatment modalities depends on the patient's chronologic and physiologic age, level of activity, bone quality, associated comorbidities, and fracture characteristics.^4^ Displaced fractures are best treated with a prosthetic replacement in elderly patients.^5^, ^6^ Fracture reduction and stable internal fixation are indicated in select groups of patients.^7^, ^8^

The importance of stable fixation in the treatment of femoral neck fractures has been emphasized, but some uncertainty remains regarding the optimal fixation method, warranting further studies.^9^, ^10^, ^11^, ^12^ One major obstacle when designing randomized clinical trials (RCTs) of hip fracture patients is the lack of accurate methods to evaluate fixation stability and fracture healing. Conventional radiography and clinical examination are thought to provide only crude estimates of the time to fracture union.^13^, ^14^ The execution of international multicenter RCTs is accompanied by additional challenges in trauma patients.^15^

Radiostereometric analysis (RSA) could help solve some of these issues in RCTs of hip fracture patients. RSA is applicable for the three‐dimensional (3D) measurement of the displacement in femoral neck fractures.^16^, ^17^, ^18^ Differentially loaded RSA (DL‐RSA) allows for the measurement of inducible micromotion under physiologic loading^19^, ^20^ but has not yet been applied in hip fracture patients. Because of its high accuracy and precision, RSA permits the minimization of sample size.^21^ This is a key benefit as patient recruitment in RCTs of hip fractures has proven challenging.^22^, ^23^

This exploratory study evaluated the feasibility of DL‐RSA in the assessment of the fracture‐site stability of internally fixed femoral neck fractures. RSA was used to measure both the degree of inducible micromotion and the permanent displacement of the fracture site.

## METHODS

The study protocol was approved by the local institutional review board and Ethical Committee of the Hospital District of Southwest Finland, Turku, Finland (decision §106, April 20, 2010) and was conducted in accordance with the Declaration of Helsinki. All participants provided written informed consent. This study was a prospective cohort study (Diagnostic Level II).

### Participants

The study population consisted of a cohort of eligible patients at a single center. A power analysis was not possible due to the exploratory nature of the study. The inclusion criteria included ambulatory men and women ≥50 years of age who presented with an isolated fracture of the femoral neck (AO/OTA types 31‐B1, 31‐B2, and 31‐B3 fractures).^24^ Patients were scheduled for surgical treatment within 48 h of presenting to the emergency room. The exclusion criteria included patients not suitable for internal fixation of the fracture (i.e., rheumatoid arthritis, severe osteoarthritis, pathologic fracture, or secondary osteoporosis including corticosteroid use).

**Figure 1:**
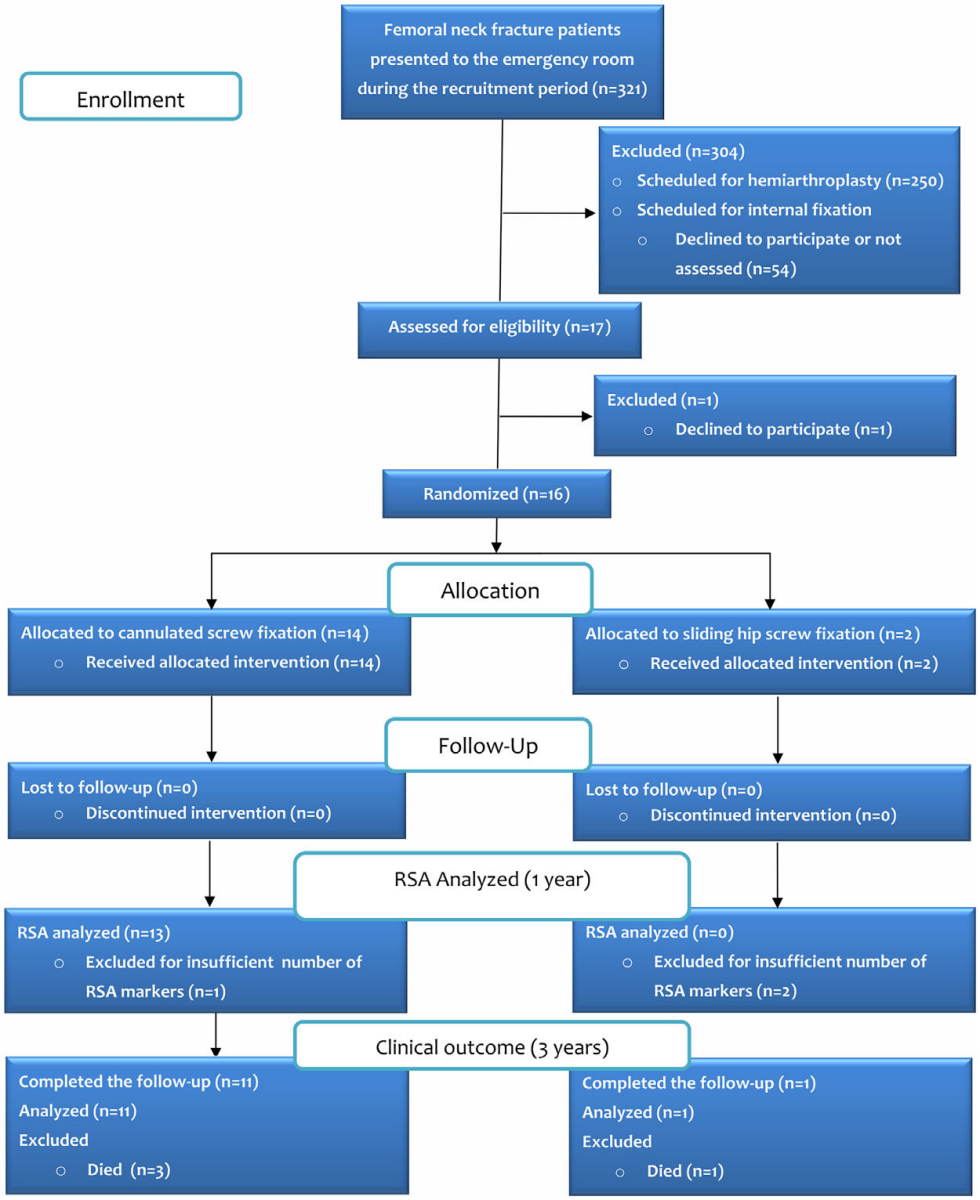
A diagram of patient recruitment and flow through the study.

During the recruitment period between September 2010 and June 2011, 321 patients were admitted for a femoral neck fracture (Fig. 1). Sixteen patients with an average age of 72 years were enrolled (Table 1). According to the protocol, patients were randomized to receive either multiple cannulated screws or a sliding hip screw (Fig. 1), but randomization was stopped early due to technical difficulties encountered in the implantation of RSA markers in two patients treated with sliding hip screw fixation.

**Table 1:** Demographics, Fracture Characteristics, and Radiographic Outcome

Case #	Gender/Age	Fracture Type	DXA T‐score	Fracture Fixation	Radiographic Outcome
1	M/59	B2	−1.6	CS	Union
2	F/75	B1,1	−3.3	CS	Union^a^
3	F/81	B1,2	−2.6	CS	Union
4	F/84	B1,2	−4.8	SHS	Union^a^
5	M/62	B2	−2.2	SHS	Union^a^
6	M/70	B1,1	−1.6	CS	Union
7	M/56	B3	−1.6	CS	Union
8	F/76	B1,1	−2.7	CS	Union
9	F/90	B1,1	−1.7	CS	Union
10	F/81	B2	−2.6	CS	Union
11	M/63	B1,1	−0.5	CS	Union
12	F/70	B1,1	−3.2	CS	Osteonecrosis
13	F/84	B1,1	−1.9	CS	Osteonecrosis
14	M/77	B2	−3.0	CS	Non‐union
15	F/72	B3	−3.9	CS	Non‐union
16	F/59	B2	−3.6	CS	Non‐union

CS, cannulated compression screws; SHS, sliding hip screw.

^a^ Excluded from the RSA analysis.

### Fracture Fixation With Cannulated Screws and Postfracture Care

Standard surgical techniques recommended by the AO Foundation were followed. Nondisplaced and valgus‐impacted 31‐B1 fractures were not reduced before fixation. The fractures were fixed with three parallel 6.5 mm cannulated compression screws. Special attention was paid to the correct placement of the screws.^25^, ^26^ Patients received preoperative infection and postoperative deep vein thrombosis prophylaxis and multimodal pain management. Patients were mobilized with the aid of rolling walkers and/or crutches. Weight‐bearing, as tolerated, was allowed without any range‐of‐motion restrictions.

### Marker Insertion and RSA Imaging

**Figure 2:**
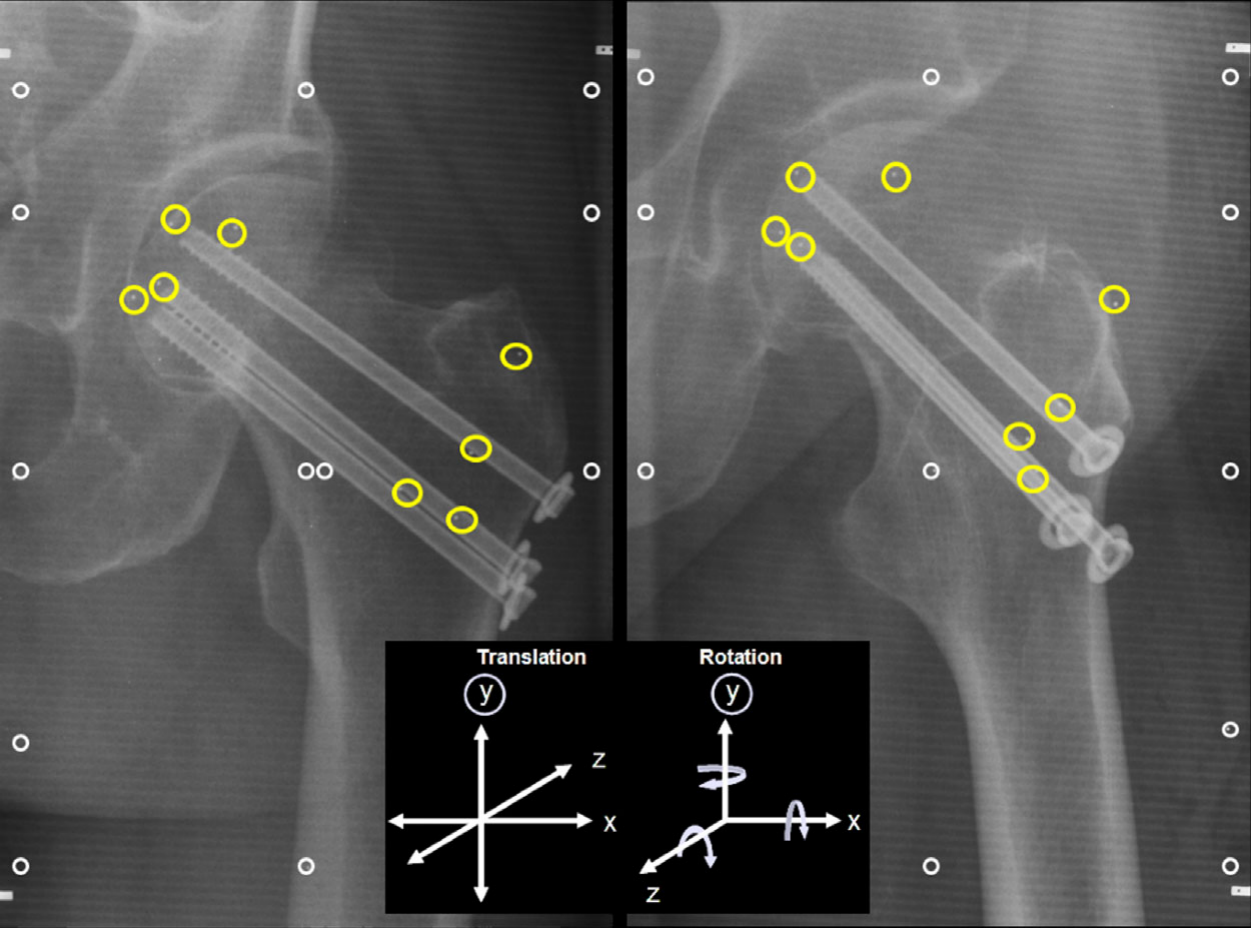
Paired RSA images (stereoradiographs) taken from a femoral neck fracture fixed with three cannulated compression screws. Yellow circles denote the locations of the inserted RSA tantalum markers in the femoral head and in the trochanteric region. Calibration cage markers are indicated with white circles. The three orthogonal axes (x, y, z) constituting the coordinate system for measuring translation and rotation are given in the black box.

During surgery, a minimum of three RSA markers (tantalum beads, ϕ 1.0 mm, Wennbergs Finmek AB, Gunnilse, Sweden) were inserted on both sides of the fracture into the femoral head and greater trochanter (Fig. 2) using an applicator (Tantalum Inserter, Wennbergs Finmek AB, Gunnilse, Sweden) under an image intensifier. Bone wax was utilized to capture a tantalum marker ball inside the distal tip of the applicator. The spring‐loaded piston of the applicator was used to fire one marker at a time into the bone. The applicator barrel was narrow enough to allow the markers to be inserted into the femoral head through the cannulated screws.

**Figure 3:**
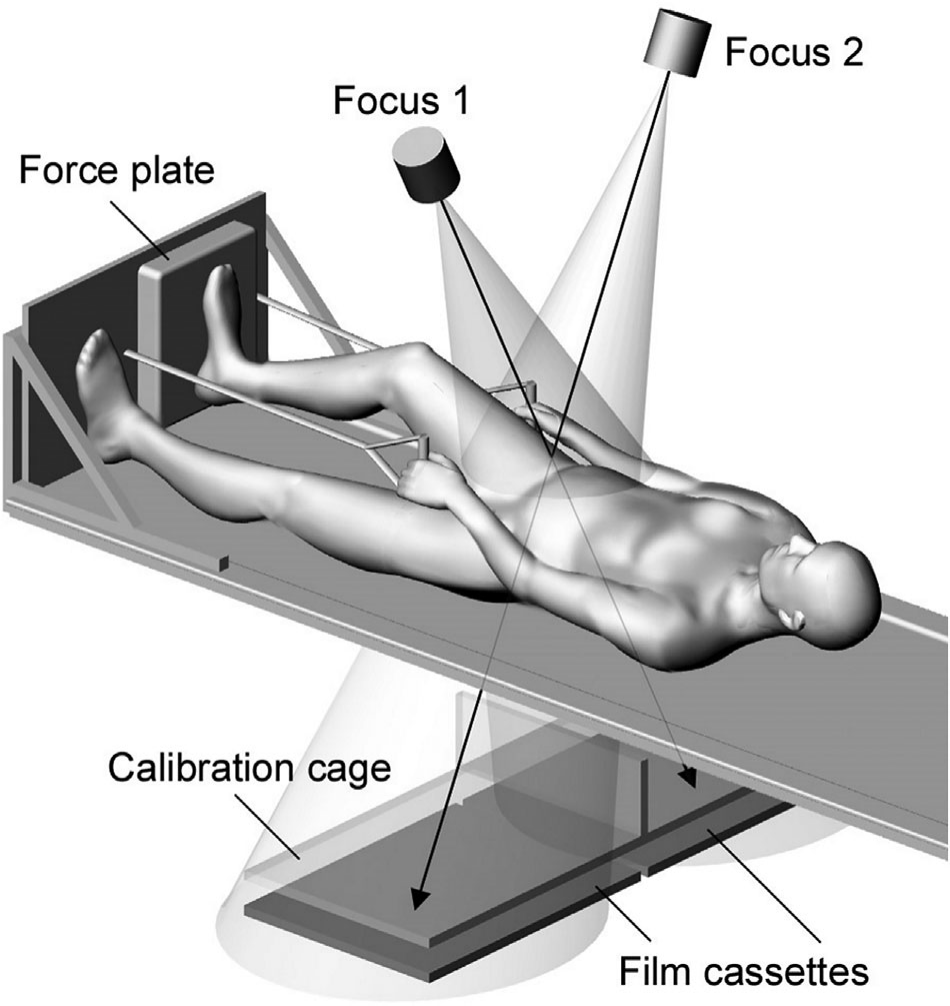
Setup for differentially loaded RSA with the uniplanar technique. Two X‐ray tubes were positioned at a 40° angle to each other in such a way that the X‐ray beams crossed at the site of the femoral neck fracture. A calibration cage, which was placed under the examination table at a fixed height, contained tantalum markers at defined positions to create a 3‐D coordinate system for calculating fracture marker displacement. The X‐ray tubes were operated simultaneously in order to obtain paired images. During differentially loaded RSA, the patient pressed a force plate as much as tolerated with the foot of the operated limb while providing a counterforce with both hands.

RSA was performed within 1–3 days after surgery (baseline) and repeated at 6, 12, 24, and 52 weeks. The patients were imaged in the supine position on the examination table (Fig. 3). The first pair of RSA images were taken without loading. The patients were then asked to press a force plate as much as tolerated with the foot of the operated limb. The rotational position of the limb and the semiflexion position of the hip was controlled. During peak loading, a second pair of RSA images were taken. The mean compression force of the two loading cycles was recorded. Patients were asked to report any pain experienced during loading on a visual analog scale of 0–10.

Image analyses were performed using UmRSA version 6.0.3.7 software (RSA BioMedical Innovations AB, Umeå, Sweden). The RSA measurement process is semiautomatic, thus minimizing subjective bias. The median number of analyzed markers was three (range 3–4) in the femoral head and four (range 3–5) in the lateral trochanteric region. The RSA markers on both sides of the fracture formed two distinct segments. At each time point, the position of the femoral head segment relative to the trochanteric segment was compared with the original position at baseline as a measure of permanent fracture‐site displacement. Unloaded and loaded images were compared in order to detect inducible 3‐D micromotion at the fracture‐site at each time point. Both permanent displacement and inducible micromotion were analyzed as linear and angular movements on three orthogonal axes (x, y, z) (Fig. 2). Left‐sided hip results were mirrored to facilitate the analysis of both sides as one group. Translation and rotation vectors were calculated to summarize total translation and rotation.^27^ According to the international RSA standardization guidelines^21^, data on individual axes are also provided (see supplemental Tables S‐1 and S‐2).

The stability and adequate distribution of the RSA markers were assessed by calculating the mean error of rigid body fitting (ME) and condition number (CN), respectively.^21^ The median ME was 0.21 (range 0.12–0.58) for the femoral head and 0.12 (range 0.07–0.22) for the reference segment (trochanter). The median CN was 113 (range 61–244) for the femoral head markers and 109 (range 57–192) for the trochanter markers. All patients were subjected to a double examination at least once, with repositioning of the patient and X‐ray tubes between examinations with and without loading. Based on these double examinations, the precision of the RSA measurements for translation and rotation was estimated as a 95% confidence interval (CI) for each axis (Table 2), as recommended.^27^

**Table 2:** Precision of RSA Measurements

	Translation (mm)				Rotation (degrees)			
RSA	x‐axis	y‐axis	z‐axis	Vector	x‐axis	y‐axis	z‐axis	Vector
Without loading	0.3	0.2	0.4	0.4	1.1	0.9	0.5	1.2
With loading	0.3	0.2	0.5	0.3	1.7	1.6	0.7	1.2

### Analysis of Plain Radiographs

Anteroposterior (AP) and lateral plane hip radiographs were taken after surgery and at each follow‐up visit. The quality of the fracture reduction was assessed based on the Garden alignment criteria.^28^ As recommended,^13^ union was defined as an asymptomatic patient with radiographic disappearance of the cortical and trabecular fracture lines. Each subject was followed clinically for up to a minimum of 3 years based on a review of the electronic chart records. Postoperative radiographs were also assessed for osteonecrosis, defined as radiographic evidence of segmental collapse of the femoral head in a symptomatic patient.

As a reference for RSA‐based measurements of permanent fracture‐site displacement, femoral neck shortening (FNS) was measured from the AP radiographs, as described previously,^29^ using a custom‐modified software. The repeatability of plain radiograph analysis was assessed through intra‐class correlation coefficients (ICC) for both intra‐observer and inter‐observer agreement between two independent investigators (SF, NM). The mean intra‐observer ICC was 0.98, and the mean inter‐observer ICC was 0.93.

### Dual‐Energy X‐Ray Absorptiometry (DXA) and Osteoporosis Treatment

The bone mineral density of the contralateral hip and lumbar spine was measured postoperatively with dual‐energy X‐ray absorptiometry (Hologic Discovery A, Hologic Inc., Marlborough, MA) (Table 1). The results were reported as the lowest T‐score. Preoperatively, two patients had received bisphosphonates for osteoporosis. After surgery, all osteoporotic patients (T‐score <−2.5) were treated with a bisphosphonate or denosumab.

### Statistical Methods

Statistical analysis was performed using SPSS version 25 (IBM SPSS Statistics, Armonk, NY). The normality of the data was assessed using the Shapiro–Wilk test. The cut‐off value for the presence of inducible micromotion was chosen based on measurement error: Inducible micromotion was considered significant when it matched or exceeded precision values of 0.3 mm for the translation vector or 1.2 degrees for the rotation vector. Analyses of mean inducible micromotion and permanent displacement were performed using repeated‐measures ANOVA. Linear regression analyses were used to identify any associations between inducible micromotion and permanent displacement as well as between the plain radiograph analysis and RSA.

## RESULTS

### Inducible Micromotion

**Figure 4:**
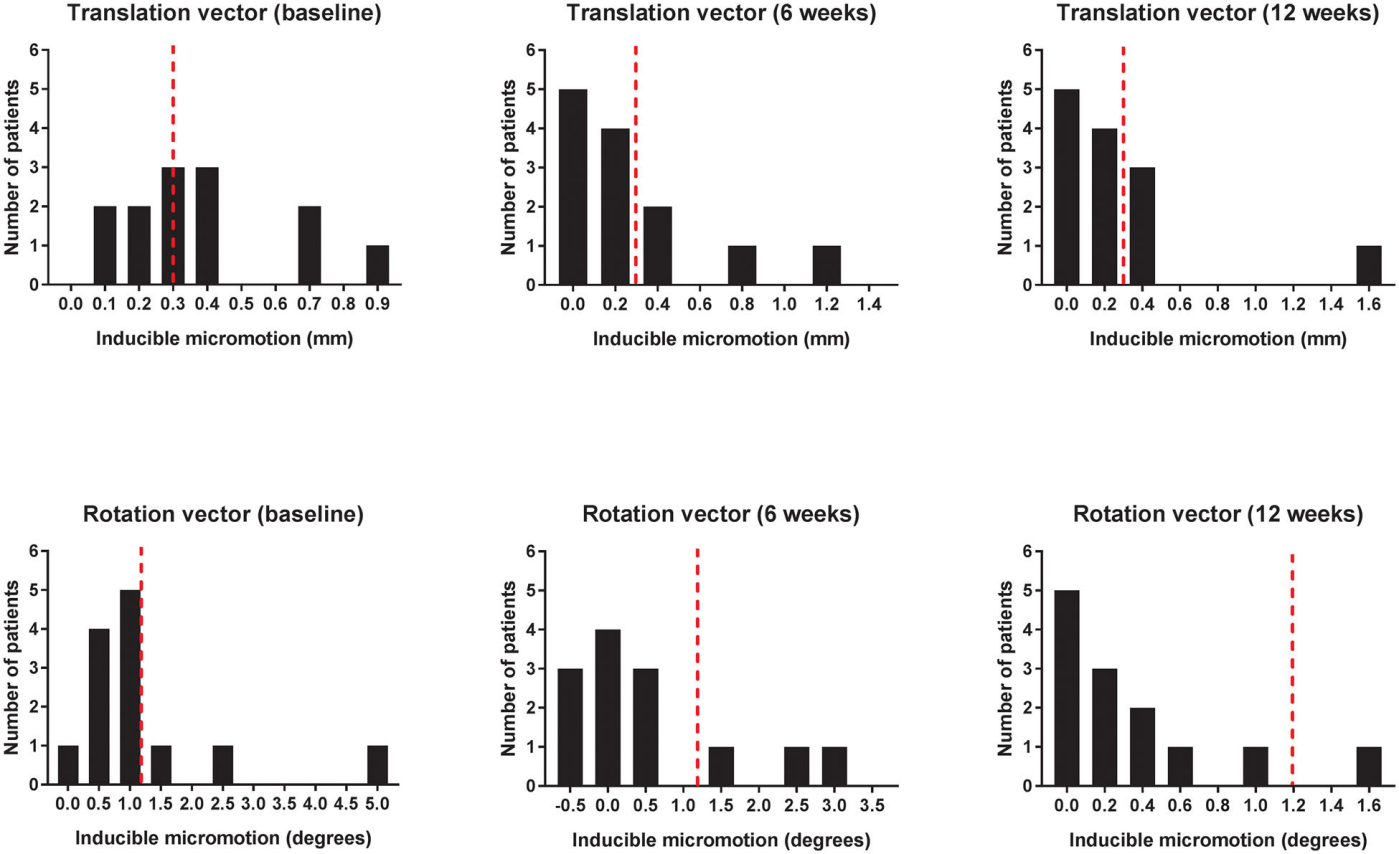
The distribution of measured inducible translation and rotation (vectors) during the first 12 weeks of healing. The number of patients with inducible micromotion gradually decreased during healing. Bars represent the number of patients. Dotted lines (red) represent the precision limits of RSA beyond which inducible micromotion was considered significant.

Voluntary loading of the operated limb (Figure 3) induced fracture‐site micromotion, which ranged from 0.39 mm (CI 0.24, 0.54) at baseline to 0.32 mm (CI 0.09, 0.54) at 6 weeks and 0.27 mm (CI 0.02, 0.52) at 12 weeks. Inducible rotation decreased from 1.17 degrees (CI 0.42, 1.91) at baseline to 0.62 degrees (CI −0.07, 1.31) at 6 weeks and 0.36 degrees (CI 0.10, 0.62) at 12 weeks. The number of patients with inducible micromotion gradually reduced from baseline to 12 weeks (Fig. 4).

At baseline, inducible translation occurred mainly in the sagittal plane (along the *z*‐axis) (Table S‐1), and translation along the *y*‐axis was minimal. Inducible rotation of the femoral head occurred mainly around the *y*‐axis at baseline (Table S‐1).

The compression force measured by the force place increased from 139 N (CI 89, 190) at baseline to 181 N (CI 129, 232) at 6 weeks and 186 N (CI 128, 243) at 12 weeks. Five patients at baseline and four patients at 6 weeks experienced moderate local pain during loading.

As a post hoc analysis, patients with and without fracture healing complications showed a significantly different (*p* = 0.029, Fisher's exact test) incidence of inducible micromotion. Patients with fracture unions were characterized by no inducible micromotion or inducible micromotion only at baseline. Patients with complications, including a patient with fracture union with malrotation (case #1, Table 1), had inducible micromotion beyond baseline (6–12 weeks). One exception was a patient with late osteonecrosis, who had micromotion only at baseline.

### Permanent Fracture‐Site Displacement

**Figure 5:**
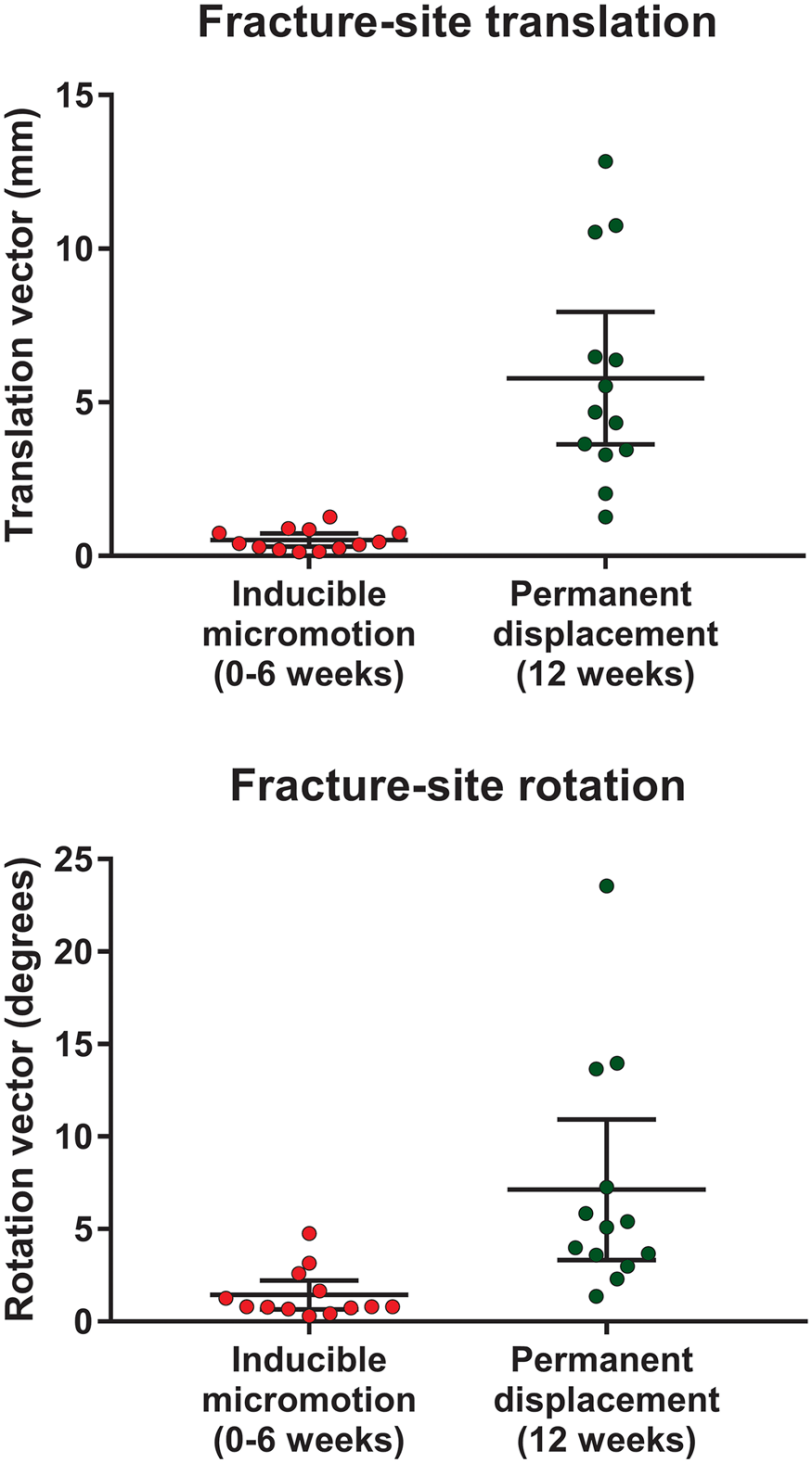
The amount of inducible fracture‐site translation and rotation was only a fraction of the magnitude of permanent fracture‐site displacement. The dots represent the translation and rotation measured in individual patients (*n* = 13). Bars represent the mean and 95% confidence interval.

Permanent fracture‐site migration was nearly an order of magnitude greater than inducible micromotion (Fig. 5). Expressed as a median percentage (interquartile range), inducible translation was 12% (12.1) and inducible rotation was 16% (19.0) from the permanent interfragmentary displacement. A regression analysis showed no significant relationship between fracture‐site displacement and inducible micromotion.

**Figure 6:**
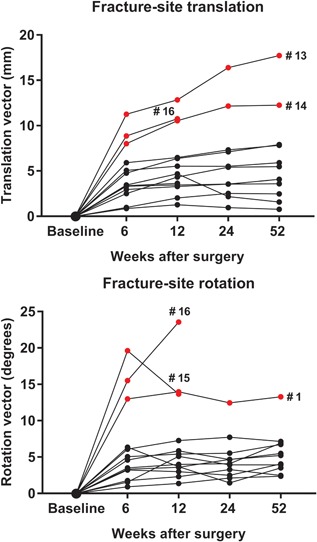
The translation and rotation of individual femoral neck fractures (*n* = 13) from the time of surgery. Outliers are marked in red. Outlier #1 had malpositioned cannulated compression screws, and outliers #13–#16 developed a nonunion or osteonecrosis.

Permanent translation occurred mainly along the *y*‐axis (axial shortening), while rotation mainly occurred around the *z*‐axis (adduction/varus) (Table S‐2). Fracture unions were characterized by the cessation of interfragmentary displacement by 12 weeks (Table 3). Patients with complications were detected as outliers in translation, rotation, or both (Fig. 6). The only exception was case #12, who developed late osteonecrosis without major fracture‐site displacement.

**Table 3:** Permanent Fracture‐Site Displacement

Time After Surgery	Translation Vector (mm)	Rotation Vector (degrees)
6 weeks	3.41 (1.88, 4.94)^a^	4.89 (1.84, 7.94)
12 weeks	4.14 (2.51, 5.77)^b^	5.49 (2.46, 8.51)
24 weeks	4.09 (2.14, 6.04)^b^	5.01 (2.30, 7.72)
52 weeks	4.26 (1.99, 6.53)^b^	5.94 (3.14, 8.73)

Mean values with 95% confidence intervals are shown (*n* = 8). Different subscript letters indicate the significant difference (*p* = 0.031 for the translation vector) (repeated‐measures ANOVA). The differences of the rotation vector not significant (*p* = 0.12).

### Analysis of Plain Radiographs and Clinical Follow‐Up

According to the Garden alignment criteria, three patients had a mild retroversion of the femoral head. Two patients had compression screws placed in the anterior part of the femoral head. Compression screws were not in direct contact with the cortical bone of the femoral neck in three patients. Fracture union was observed in eleven patients (69%). Osteonecrosis developed in two patients. Of those patients with a nonunion or osteonecrosis (#12‐16, Table 1), two underwent early total hip replacement, one patient had a late total hip replacement, and two patients underwent only implant removal.

### Plain Radiograph Measurements and Correlation to RSA Results

Based on the 52‐week AP radiographs of 14 patients (excluding the two patients who required an early hip replacement), the shortening of the abductor moment arm (denoted by the *x*‐axis) was moderate (5‐10 mm) in four cases and severe (>10 mm) in one case. Femoral length reduction (denoted by the *y*‐axis) was moderate (5–10 mm) in two cases and severe (>10 mm) in seven cases. The FNS vector along the axis of the femoral neck shaft (denoted by the *z*‐axis) was 9.2 mm (CI 5.7, 12.7).

The RSA‐measured 3‐D fracture‐site displacement correlated with the two‐dimensional radiographic measurements of postfracture femoral neck deformity. The RSA‐measured translation vector had a high coefficient of determination (*R* ^2^ = 0.797, *p* < 0.001) with the FNS vector measured from plain radiographs. RSA‐measured translations along the *x*‐axis and the *y*‐axis also had high coefficients of determination (*R* ^2^ = 0.700, *p* = 0.001 and *R* ^2^ = 0.625, *p* = 0.004) with the decrease in the abductor moment arm and femoral length, respectively.

## DISCUSSION

Stable internal fixation achieved with static compression of the fracture surfaces plays a key role in the treatment of femoral neck fractures.^4^, ^8^ This is in contrast with diaphyseal long‐bone fractures, where cyclic fracture‐site micromotion is a desirable phenomenon to stimulate callus formation.^30^ Although stability is the primary goal of treatment, in vivo data on the stability of femoral neck fractures stabilized under internal fixation is still scarce. This is due to the lack of suitable research tools to assess initial fracture‐site stability. Our exploratory study was designed to examine the feasibility of DL‐RSA for this purpose. Our results suggested that DL‐RSA can detect the presence of inducible micromotion in internally fixed femoral neck fractures. Detecting inducible micromotion beyond baseline might serve as a dichotomous measure of initial fixation instability.

Micromotion exceeding the precision values of RSA was used as the criterion for inducible micromotion. Inducible micromotion values had a dichotomous distribution. The subgroup of patients with uncomplicated fracture unions had no inducible micromotion or micromotion only at baseline, while failure cases tended to have inducible micromotion beyond baseline and were outliers in permanent fracture‐site displacement. These observations suggest the clinical relevance of DL‐RSA measurements and support the validity of our technique for defining significant inducible micromotion.

Our previous study that used the group mean of inducible micromotion as an outcome measure seemed to overestimate the incidence and timeframe of the motion of distal radial fractures treated with a volar plate.^20^ One possible explanation is that the plasticity of a uniting fracture callus may permit enough motion to be detectable with RSA on group‐level. This further emphasizes the relevance of the dichotomous classification of significant micromotion in DL‐RSA on individual‐level. Our study was not powered for defining any safety limits or cutoff points for fracture‐site micromotion, but considering our clinical results, it seems that our choice of criteria for inducible micromotion was relevant.

Confirming the results of in vitro biomechanical studies of cadaver femurs,^31^ the current study demonstrated that compression screws permit fracture‐site micromotion under loading. This observation supports the assertion that our clinical DL‐RSA setup generated sufficient loading at the hip. Indeed, clinical studies with instrumented hip prostheses have demonstrated that simple dynamic exercises in the supine position generate high hip loads.^32^

Despite the small sample size, the clinical outcome of our study population was in line with previously published literature. The percentage of fracture unions (69%, Table 1) resembled previously published internal fixation success rates.^33^, ^34^ The results of our AP radiograph analysis were also in line with studies with similar study populations.^29^, ^35^

Unexpectedly, but in line with the results of a recent study,^18^ we encountered technical difficulties in the implantation of RSA markers into the femoral head. The original RSA technique described for femoral neck fractures involved the use of predrilled bone channels.^36^ In the current study, cannulated compression screws allowed for the easy atraumatic implantation of markers, while the technique we applied to patients treated with a sliding hip screw was not successful. Using the applicator under image intensification, it was difficult to insert multiple markers through the joint capsule into optimal anatomic locations in the femoral head. It also became evident that in repeated attempts the applicator could create unwanted, albeit narrow, bone channels in the head. The technique also carries a risk for extraskeletal loose beads near the joint cavity. We, therefore, concluded that an unbiased comparison of the RSA of the two fixation methods was not possible to achieve, and the randomization of patients according to the fixation method was terminated. Future RSA studies of sliding hip screws should explore the use of cannulated lag and antirotation screws for marker insertion. Additional markers will still need to be inserted through some other route. Without a doubt, any novel technique should be tested in a phantom model, as recommended,^21^ to ensure feasibility, appropriate marker scatter and safety.

As a limitation, the cannulated screws permitted the implantation of only three RSA markers into the femoral head, which is the minimum requirement for this method. As demonstrated in a model of tibial plateau fractures,^37^ increasing the number of RSA markers would significantly increase the accuracy and precision of our technique. The calculated precision thresholds for inducible micromotion (0.3 mm and 1.2 degrees)(Table 2) were higher than those (0.1 mm and 1.0 degrees) observed in our previous study of plated distal radial fractures that used a higher number of RSA markers in both segments (*n* ≥ 4–5).^20^ Our precision thresholds for permanent fracture‐site displacement (0.4 mm and 1.2 degrees) were also higher than those reported in a phantom model of trochanteric fractures (0.1–0.3 mm and 0.5 degrees), which allowed for an ideal spatial implantation of multiple markers.^38^ As a related issue, the spatial distribution of the markers that we achieved was not always optimal. RSA computer programs calculate a condition number as an objective indicator of appropriate marker scatter. High condition numbers indicate poor marker distribution; an upper limit of 150 has been recommended.^21^ However, in line with previous RSA studies of small bones and joints, we accepted condition numbers above 150.^20^, ^39^ The precision of the measurements was therefore validated in each patient with the double examination, as recommended.^21^ Finally, we recognize that the small number of participants limits the clinical conclusions that can be drawn from the results. Our study was not powered to answer fundamental questions about the optimal rigidity of fracture fixation or the role of guided fracture‐site impaction for successful clinical outcomes. It should be noted that RSA is a demanding research method that is currently restricted to clinical trials alone and not for routine clinical practice.

## AUTHORS' CONTRIBUTIONS

SF gathered data, performed the plain radiograph analysis and helped write the manuscript. NM contributed to the design of the study, performed the RSA and aided in the plain radiograph analysis. NS helped with the clinical execution of the study. JJA helped with data collection and writing the manuscript. HA designed the study, executed the clinical trial, analyzed data and prepared the manuscript. All authors have read and approved the final submitted manuscript.

## Supporting information

Additional supporting information may be found online in the Supporting Information section at the end of the article.

Supporting Table S1.

Supporting Table S2.
