# Supplementary material for: Radiostereometric analysis of the initial stability of internally fixed femoral neck fractures under differential loading
Source: J Orthop Res. 2018 Oct 25;37(1):239–47. doi: 10.1002/jor.24150 (PMC6587786; doi:10.1002/jor.24150)
Supplement: Supplementary file 1 — Supporting Table S1. [file JOR-37-239-s001.docx]

**Table S-1.** Inducible Micromotion

| Time after  surgery  (weeks) | Lateral-medial  x-axis translation,  mm | Distal-proximal  y-axis translation,  mm | Posterior-anterior  z-axis translation,  mm |
| --- | --- | --- | --- |
| 0 | -0.00 (-0.09, 0.09 ) | -0.06 (-0.13, 0.02) | 0.21 (-0.02, 0.43) |
| 6 | 0.11 (-0.02, 0.23) | -0.32 (-0.62, -0.02) | 0.01 (-0.33, 0.34) |
| 12 | 0.05 (-0.07, 0.17) | -0.34 (-0.70, 0.03) | -0.02 (-0.27, 0.23) |

| Time after  surgery  (weeks) | Anterior tilt  x-axis rotation,  degrees | Internal rotation  y-axis rotation,  degrees | Adduction  z-axis rotation,  degrees |
| --- | --- | --- | --- |
| 0 | -0.03 (-0.59, 0.53) | 0.51 (-0.25, 1.27) | -0.13 (-0.44, 0.18) |
| 6 | -0.76 (-1.90, 0.38) | -0.01 (-0.37, 0.34) | -0.28 (-0.56, -0.01) |
| 12 | 0.21 (-0.45, 0.87) | 0.17 (-0.27, 0.61) | -0.34 (-0.66, -0.02) |

Mean values with 95% confidence intervals are shown (n = 13)
