# Supplementary material for: Radiostereometric analysis of the initial stability of internally fixed femoral neck fractures under differential loading
Source: J Orthop Res. 2018 Oct 25;37(1):239–47. doi: 10.1002/jor.24150 (PMC6587786; doi:10.1002/jor.24150)
Supplement: Supplementary file 2 — Supporting Table S2. [file JOR-37-239-s002.docx]

**Table S-2.** Permanent Fracture-Site Displacement

| Time after  surgery  (weeks) | Lateral-medial  x-axis translation,  mm | Distal-proximal  y-axis translation,  mm | Posterior-anterior  z-axis translation,  mm |
| --- | --- | --- | --- |
| 6 | -0.75 (-1.61, 0.10) | -3.32 (-5.04, -1.60) | -1.04 (-2.07, -0.02) |
| 12 | -1.00 (-2.21, 0.21) | -4.10 (-6.07, -2.13) | -1.06 (-2.27, 0.16) |
| 24 | -1.57 (-3.49, 0.35) | -5.34 (-8.42, -2.25) | -1.58 (-3.32, 0.17) |
| 52 | -1.52 (-3.43, 0.38) | -5.54 (-8.96, -2.13) | -1.80 (-3.72, 0.13) |

| Time after  surgery  (weeks) | Anterior tilt  x-axis rotation,  degrees | Internal rotation  y-axis rotation,  degrees | Adduction  z-axis rotation,  degrees |
| --- | --- | --- | --- |
| 6 | -0.93 (-4.48, 2.63) | 0.75 (-1.36, 2.86) | -2.65 (-5.01, -0.28) |
| 12 | -0.84 (-3.41, 1.72) | 0.88 (-1.41, 2.89) | -3.27 (-6.04, -0.49) |
| 24 | 0.12 (-1.56, 1.79) | 0.23 (-1.96, 2.41) | -4.20 (-7.40, -1.00) |
| 52 | -0.08 (-2.20, 2.05) | 0.01 (-3.06, 3.07) | -4.52 (-7.86, -1.17) |

Mean values with 95% confidence intervals are shown (n = 13)
